# Impact of nanophos in agriculture to improve functional bacterial community and crop productivity

**DOI:** 10.1186/s12870-021-03298-7

**Published:** 2021-11-08

**Authors:** Parul Chaudhary, Anuj Chaudhary, Heena Parveen, Alka Rani, Govind Kumar, Rajeew Kumar, Anita Sharma

**Affiliations:** 1 Department of Microbiology, College of Basic Sciences and Humanities, G.B. Pant University of Agriculture and Technology, Pantnagar, Uttarakhand India; 2 School of Agriculture and Environmental Sciences, Shobhit University, Gangoh, Uttar Pradesh India; 3 Department of Microbiology, Gurukula Kangri Vishwavidyalaya, Haridwar, Uttarakhand India; 4 Crop Production Division, Central Institute for Subtropical Horticulture, Lucknow, Uttar Pradesh India; 5 Department of Agronomy, College of Agriculture, G.B. Pant University of Agriculture and Technology, Pantnagar, Uttarakhand India

**Keywords:** Maize, Nanophos, NPK solubilizers, Protein, Soil enzyme activities

## Abstract

**Background:**

Since the World’s population is increasing, it’s critical to boost agricultural productivity to meet the rising demand for food and reduce poverty. Fertilizers are widely used in traditional agricultural methods to improve crop yield, but they have a number of negative environmental consequences such as nutrient losses, decrease fertility and polluted water and air. Researchers have been focusing on alternative crop fertilizers mechanisms to address these issues in recent years and nanobiofertilizers have frequently been suggested. “Nanophos” is a biofertilizer and contains phosphate-solubilising bacteria that solubilises insoluble phosphate and makes it available to the plants for improved growth and productivity as well as maintain soil health. This study evaluated the impact of nanophos on the growth and development of maize plants and its rhizospheric microbial community such as NPK solubilising microbes, soil enzyme activities and soil protein under field condition after 20, 40 and 60 days in randomized block design.

**Results:**

Maize seeds treated with nanophos showed improvement in germination of seeds, plant height, number of leaves, photosynthetic pigments, total sugar and protein level over control. A higher activity of phenol, flavonoid, antioxidant activities and yield were noticed in nanophos treated plants over control. Positive shift in total bacterial count, nitrogen fixing bacteria, phosphate and potassium solubilizers were observed in the presence of nanophos as compared to control. Soil enzyme activities were significantly (*P* < 0.05) improved in treated soil and showed moderately correlation between treatments estimated using Spearman rank correlation test. Real time PCR and total soil protein content analysis showed enhanced microbial population in nanophos treated soil. Obtained results showed that nanophos improved the soil microbial population and thus improved the plant growth and productivity.

**Conclusion:**

The study concluded a stimulating effect of nanophos on *Zea mays* health and productivity and indicates good response towards total bacterial, NPK solubilising bacteria, soil enzymes, soil protein which equally showed positive response towards soil nutrient status. It can be a potential way to boost soil nutrient use efficiency and can be a better alternative to fertilizers used in the agriculture.

**Supplementary Information:**

The online version contains supplementary material available at 10.1186/s12870-021-03298-7.

## Background

Present agricultural practices face major challenges like decline in agricultural productivity and deterioration in sustainability of agro-ecosystem worldwide. Food production cannot be sustainable unless a soil has a sufficient and proactive microbial population [1]. Maize (*Zea mays* L.) is the major food globally and known as the queen of cereal crops. However, agricultural yield of major crops is severely low due to injudicious application of agrochemicals and nutrient insufficiency that rigorously affects overall plant and soil health. Thus, there is a need to focus on good quality agronomic practices.

Agricultural sector is growing progressively with the introduction and implementation of innovative tools and techniques such as nanomaterials, nanofertilizer and nanobiofertilizers. Nanotechnology is an innovative field of science with applications in a variety of fields, including food and agriculture [2]. Nano materials having high surface tension holds the material more strongly than conventional surfaces thus helps in slow release of fertilizers makes them useful in agricultural sector [3]. Combined application of bioformulated plant useful microorganisms and nanocompounds were traditionally used as an effective replacement for chemical fertilizers in ancient times.

Phosphorus is essential nutrients required in adequate amount by plants but present in unavailable form. It is required for proper plant growth and development. Plants could enhance P uptake from the soil by varying root morphology [4]. Physiological changes like release of organic acids, release of protons, phenolics, phytase and phosphatase enzymes increase the P availability in the rhizosphere. Therefore, phytase and phosphatase enzymes released by microbes and roots help in mineralization of P and easily available to plants. Phosphorus solubilising microbes (PSM) is microbial fertilizers with broad prospective and nanophos in form of liquid formulation helps in root proliferation and controls soil borne pathogens. It also increases the capacity of the plant for up taking of nutrients, increases the seed germination, maturity ratio of grain and yield.

Importance of bioformulations using bioinoculants in agricultural sector is well known [5]. They assist in nutrient acquisition through nitrogen fixation, siderophore production, Hydrogen cyanide production (HCN) and phosphate solubilization thus supporting plant growth [6, 7]. PGPR have been studied and many biofertilizers are commercialized in the market such as the species *Pseudomonas*, N fixing *Azospirillum* and *Bacillus* [8]. There are various PSB such as *Pseudomonas taiwanensis*, *Pantoea agglomerans*, *Bacillus* and *Sinorhizobium* [9]*.* However, widespread application of biofertilizers by farmers is still limited. Certain challenges are associated with product development under optimized lab conditions to their wide application in fields like inconsistency of PGPR and inadequate shelf life of bioinoculants [10]. Nanoencapsulation of biofertilizers using natural polymers could be used as a versatile tool in enhancing the shelf life of PGPR [11]. In addition, nanoparticles assist in controlled release of nutrients that prevents leaching and evaporation of harmful substances. This in turn maintains the soil fertility and useful soil microflora. PGPB can be applied on seeds by various formulations (liquid, organic and inorganic) and influence the bio stimulant effectiveness [12].

Soil microbes such as PSB and nitrogen fixing bacteria help in restoration of soil fertility by nutrients cycling and ultimately help in plant growth [13]. Application of *Rhizobium* and *Stenotrophomonas maltophila* improved the plant growth due to increased P availability under saline condition [14]. PSB have been used as a biofertilizers in various crop such as maize, cabbage and sugarcane [15, 16]. Soil enzymes, microbial population and micro/macronutrients determined the functions of microbial community and have huge impact in maintaining soil fertility. Soil enzymes reveal the changes in soil biochemical processes and deviation in soil due to environmental factors. Estimation of soil enzyme activities in nutrient cycling has been extensively used as indicator to find out any changes by environmental/human issues [17]. These enzymes are derived from plant root exudates, animal residues and soil microbes. They play a vital role in catalysing reactions required for nutrient cycling, organic matter decomposition and stabilize soil structure. FDA, dehydrogenase, amylase and alkaline phosphates enzymes are involved in different nutrient cycling in soil such as nitrogen, carbon and phosphorus. Phosphatases catalyse the ester and anhydride hydrolysis and release free phosphate in the soil. Inoculation of biofertilizers improved the sugarcane yield, acid phosphatase activity and P content in soil over control [18].

This study was conducted to assess the impact of nanophos on maize plants growth, productivity and soil fertility under field condition. The main objectives of the study were to assess the impact of nanophos on different growth parameters of maize plants like seed germination, plant height, chlorophyll, carotenoid, protein, sugar, phenolics content, yield of maize and soil health parameters like NPK bacterial count, soil enzymes activities and evaluate bacterial population of maize rhizosphere using qPCR and soil microbial protein.

## Material and methods

### Experimental design

Field trial was conducted in the month of June to September, (2017) at Norman E Borlogue Crop Research Centre, Govind Ballabh Pant University of Agriculture & Technology, Pantnagar. Experimental section lies about 30 Km Southward of Shivalik Himalayas (location 79.3^0^ E and 29^0^N latitude). Summers are hot and warm in this area with maximum 35 °C temperature in month of July and minimum 23 °C during month of September. Relative humidity was maximum in month of July and lowest in June. Soil of the experimental site was silty clay type having the (0.206 dS/m) Electrical conductivity, Organic carbon (0.78%), nitrogen, phosphorus, potassium (215.79, 27 and 136 Kg/ha) and having pH 7.4 [19]. Total two treatments: control (without nanophos) and nanophos only were used with three replications each in randomized block design. Each plot has 4.2 m length and 3.5 m width with plant-to-plant distance was 20 cm and row to row was 60 cm.

### Seed priming with nanophos

Nanophos used in this study were provided by Department of Agronomy, GBPUA&T, Pantnagar. It is unique liquid formulation in combination with nanophosphorus and phosphate solubilising bacteria. High yielding seed variety (*Zea mays* L. cv. DH296), was obtained from Crop Research Centre, GBPUA&T. Before sowing for germination, maize seeds were properly sterilized using 70% ethanol, followed by 3% hydrogen peroxide. Seeds were washed five times with distilled water and further treated with Nanophos. Viable count for liquid formulation was 1 × 10^8^ cells/ml of liquid. Four hundred fifty microlitres nanophos was added in 5 ml distilled water and maize seeds were dipped. After proper treatment, seeds were shade dry for 10–15 min. Treated seeds were used further for field experiment [20]. Control did not receive any treatment.

### Seed germination assay

The seed germination efficacy was tested on *Zea mays* seeds using the formula:

$$\mathrm{Germination}\%\kern0.5em =\frac{\mathrm{Number}\ \mathrm{of}\ \mathrm{seedlings}\ \mathrm{germinated}}{\mathrm{Total}\ \mathrm{number}\ \mathrm{of}\ \mathrm{seeds}}\times 100$$

### Growth measurement

Average plant height, root length and number of leaves were measured after 20, 40 and 60 days of the experiment to check the effect of nanophos on growth profile of *Zea mays*.

### Estimation of total chlorophyll and carotenoid content

Photosynthetic pigment in treated leaf samples were estimated through Dimethylsulfoxide (DMSO) method given by Hiscox and Israelatum [21]. After harvesting, maize leaves were collected, washed with distilled water and finely chopped for experiment. For chlorophyll extraction 500 mg leaves were mixed with 10 ml of DMSO. Then, tubes were properly incubated at 60 °C for 2 h in a water bath. Supernatant with extract were pooled and absorbance were taken at 645 and 663 nm. Same treated extract was used for measuring carotenoid content by taking absorbance at 470mn [22].

### Total sugar content

Method given by Dubois et al. [23] was used to estimate total sugar in maize leaves using glucose as a standard curve. Dried leaves were crushed and added to 3 ml of ethyl alcohol (80%) and placed in boiling water bath. Homogenate was centrifuged at 1000 rpm for 15 min. Supernatant (1 ml) was transferred in a test tube and Anthrone reagent (4 ml) was added and placed in boiling water for 10 min. Absorbance of each sample was taken at 620 nm and total sugar was extrapolated against glucose standard curve.

### Estimation of total protein

Crushed leaves (500 mg) were homogenized with 5 ml Tris Cl (0.2 M) and centrifuged at 10,000 g for 15 min at 4 °C. Extracted supernatant (20 μl) was taken and 300 μl of double distilled water was added to bring up the volume. Bradford dye was added to the tubes and properly mixed by vortex. After incubation for few minutes absorbance was recorded at OD 595 nm against a blank (100 μl of extraction buffer with 1 ml of reagent dye) in a spectrophotometer [24].

### Determination of total phenolic content in maize leaves

Total phenolics in treated leaf samples were determined by Folin- Ciocalteu reagent by following method of Ainsworth and Gillespie [25]. Leaf extract (0.5 ml) was mixed with 2 ml of FC reagent. Mixture was incubated for 30 min at 37 °C till blue colour develops. The reading of the resultant blue colour was measured at 765 nm. Gallic acid was taken for making standard.

### Catalase activity

Leaf samples from different treatments were homogenized with 5 ml phosphate buffer (100 mM; pH 7.5). Extract was centrifuged at 4 °C for 10 min at 12,000 rpm. Supernatant was further used for enzyme assay. Assay mixture used for the experiment contained phosphate buffer and 0.1 ml of 10 mM H_2_O_2_ and 100 μl of enzyme extract.CAT activity was observed through decline in absorbance at 230 nm for 3 min corresponding to the decomposition of H_2_O_2_ [26].

### Peroxidase (POD) activity

Enzyme activity was performed using the method given by Mali et al. [27]. For peroxidase activity, 0.1 ml of enzyme extract was added to the assay mixture. The assay mixture contained 0.5 ml H_2_O_2_ and pyrogallol (0.4 ml) prepared in phosphate buffer. Peroxidase activity was measured by observing vary in absorbance at 420 nm and calculated using the extinction coefficient of 26.6 mM^− 1^ cm^− 1^.

### Superoxidase activity (SOD)

Enzyme activity was assessed by using methionine (200 mM), 75 mM riboflavin, phosphate buffer (100 mM, pH -7.5) and enzyme extract (100 μl). The activity of the SOD enzyme was by inhibition of NBT and estimated spectrophotometrically at 560 nm [28].

### Collection of soil samples

Maize plants were collected and gently shaken to remove maize rhizospheric soil (1-15 cm depth) for further experiment after 20, 40 and 60 days of sowing. Soil samples (10 g) from each replicate was taken and kept in sterilized polythene bags. Final sample were prepared by mixing individual samples after homogenization. Soil samples were sieved and used for evaluating chemical and physical properties and indicator enzymes of the soil and stored at − 20 °C for further analysis.

### Enumeration of total bacteria, nitrogen fixers, phosphorus and potassium solubilizers in treated soil

Bacterial count was checked using different types of media such as nutrient agar for total bacterial count, Ashby agar for nitrogen fixing bacteria, Aleksandrow agar for potassium solubilizers and Pikovaskaya agar for P solubilising bacteria. Plates were incubated for 2–4 days at 30 °C. Colony Forming Unit (CFU) was measured using pour plating method.

### Soil health indicator enzymes

#### Fluorescein diacetate hydrolytic (FDA) activity

One gram of soil, 50 ml sodium phosphate buffer (pH -7.6), 0.5 ml FDA solution were as added in a flask and kept for 1 h at 24 °C in incubator shaker. To stop the reaction, 2 ml of acetone was added. For 5 min, the soil suspension was centrifuged at 8000 rpm. Filter paper No.2 was used to filter the supernatant. FDA hydrolysis was assessed at 490 nm and represented as ug fluorescein g^− 1^ dry soil h^− 1^ [29].

#### Dehydrogenase activity in soil

An important soil health indicator enzyme dehydrogenase was estimated as per the method given by Casida et al. [30] using 2,3,5-Triphenyl tetrazolium chloride (TTC) substrate. Briefly, TTC substrate with pH 7.4 was added to soil sample and incubated in rotatory shaker. Reaction was allowed and terminated at different time intervals using 25 ml acetone. Suspension was centrifuged at 4000 rpm for 15 min at 4 °C and filtered using Whatman filter paper no.1. Production of dehydrogenase in soil samples were quantified by measuring insoluble product red product Triphenylformazan (TPF) formed during the reaction. Red coloured TPF can be quantified at the range of visible light (485 nm).

#### Alkaline phosphatase activity

Soil phosphatase activity in the experimental soil was performed using p- nitrophenyl phosphate as per method given by Tabatabai and Bremer [31]. Briefly, soil sample was added to 250 μl toluene followed by addition of universal buffer (100 mM, pH 11). To this solution, 1 ml Triphenylformazan (TPF) was added and complete solution was incubated at 37 °C for further reaction. After incubation for 1 h reaction was terminated using Tris buffer (pH 12, 0.1 M) and 1 ml CaCl_2_. Reaction mixture was filtered. Alkaline phosphatase was quantified by taking absorbance of product p- Nitrophenol (PNP) at 400 nm.

#### β-glucosidase activity

In a test tube, 0.25 mL toluene, 1 mL p-nitrophenyl-D-glucoside (pNPBG), 4 ml adjusted universal buffer with pH 6.0 were applied to 1 g dry soil. Tubes were incubated at 37 °C for 1 h. In a test tube, Tris buffer (pH- 12) and CaCl_2_ (1 ml, 0.5 M) were added. Spectrophotometer set at 410 nm was used to determine the intensity of the colour developed in the soil suspension. g pNP g^− 1^ dry soil h^− 1^ was used to test enzyme activity [32].

#### Amylase activity

One gram of soil sample was taken in a tube, phosphate buffer (2.5 ml; pH 6) and starch (1%, 1 ml) was added. Tubes were kept at 30 °C for 6 h in incubator shaker and centrifuged at 12000 rpm for 10 min. Supernatant (1 ml) were taken in another tube and DNS (1 ml) was added and placed in water bath at 90 °C for 5 min. Intensity of coloured product was measured by taking the readings at 540 nm [33].

#### Arylesterase

One gram soil was taken in a test tube MUB (2 ml) and 200 mM pNPA (0.5 ml) were added. Tubes were placed in water bath with constant shaking for 1 h and centrifuged at 6500 rpm for 5 min at 4 °C. Supernatant (1 ml) was taken in another tube and n-hexane (2 ml) was added. 0.5 ml of aqueous layer was taken; 0.5 ml NaOH and ddw (4 ml) were added. Absorbance was taken at 400 nm [34].

#### Quantative PCR (qPCR) analysis of 16S rDNA

Soil DNA was extracted from different rhizospheric soil samples. One gram soil was used for DNA extraction using Soil DNA Purification Kit (HiMedia). Soil DNA was quantified and purity was checked in a NanoDrop spectrophotometer at 260 and 280 nm. qPCR was performed in iCycler iQ™ Multicolor instrument. Universal primers (EUB 341F- 5′ CCTACGGGAGGCAGCAG 3′ and EUB 534R- 5′ ATTACCGCGGCTGCTGG 3′) was used to carry out real-time PCR to quantify 16S rDNA in the extracted soil DNA [35]. Total volume of qPCR reaction was 25 μl containing, 0.5 μl of individual primer, SYBR green (12.5 μl) supermix and 1 μl of soil DNA (10 ng). Melt curve analysis of 16S rDNA amplicons was also performed at the end of the q-PCR to ensure the amplification of 16S rDNA during real-time quantification. 

#### Soil protein extraction

One gram soil from different samples were incubated in nutrient broth (100 ml) for 24 h at 27 °C and then centrifuged for 10 min at 10,000 rpm. Pellet was washed in Tris/HCl with pH: 6.8 and 200 μl Tris buffer (pH 6.8) and then centrifuged for 5 min. Supernatant was discarded and pellet was dissolved in 200 μl extraction buffer and boiled for 20–30 min in water bath. Samples were centrifuged at 5000 rpm and supernatant was used for further studies.

#### SDS PAGE (sodium dodecyl sulphate-polyacrylamide gel electrophoresis)

SDS PAGE performed by using 12% resolving gel and stacking gel (4%). Protein samples for different treatments were ready in extraction buffer. Page Ruler prestained protein ladder was used as a molecular marker. The protein gel after sample loading were run using Tris glycine buffer for 6 h at 100 V. Gel was fixed for 30 min with methanol and glacial acetic acid. Gel was stained with CBBR-250, glacial acetic acid (10%) and methanol (10%) with mild shaking overnight. Gel was detained for sometimes with methanol and glacial acetic acid [36].

### Statistical analysis

Results were statistically analysed through Two Way Analysis of Variance (ANOVA) at *p* < 0.05 using SPSS software. The values of above parameters were expressed as mean ± SD (standard deviation). Distance measure in different treatments using Heatmap with Spearman rank correlation test.

## Results

### Seed germination assay

Maize seeds treated with nanophos positively influenced the seed germination rate. Treated seeds showed higher (95%) seed germination rate significantly high as compared to control (80%) respectively.

### Plant growth parameters

**Fig. 1:**
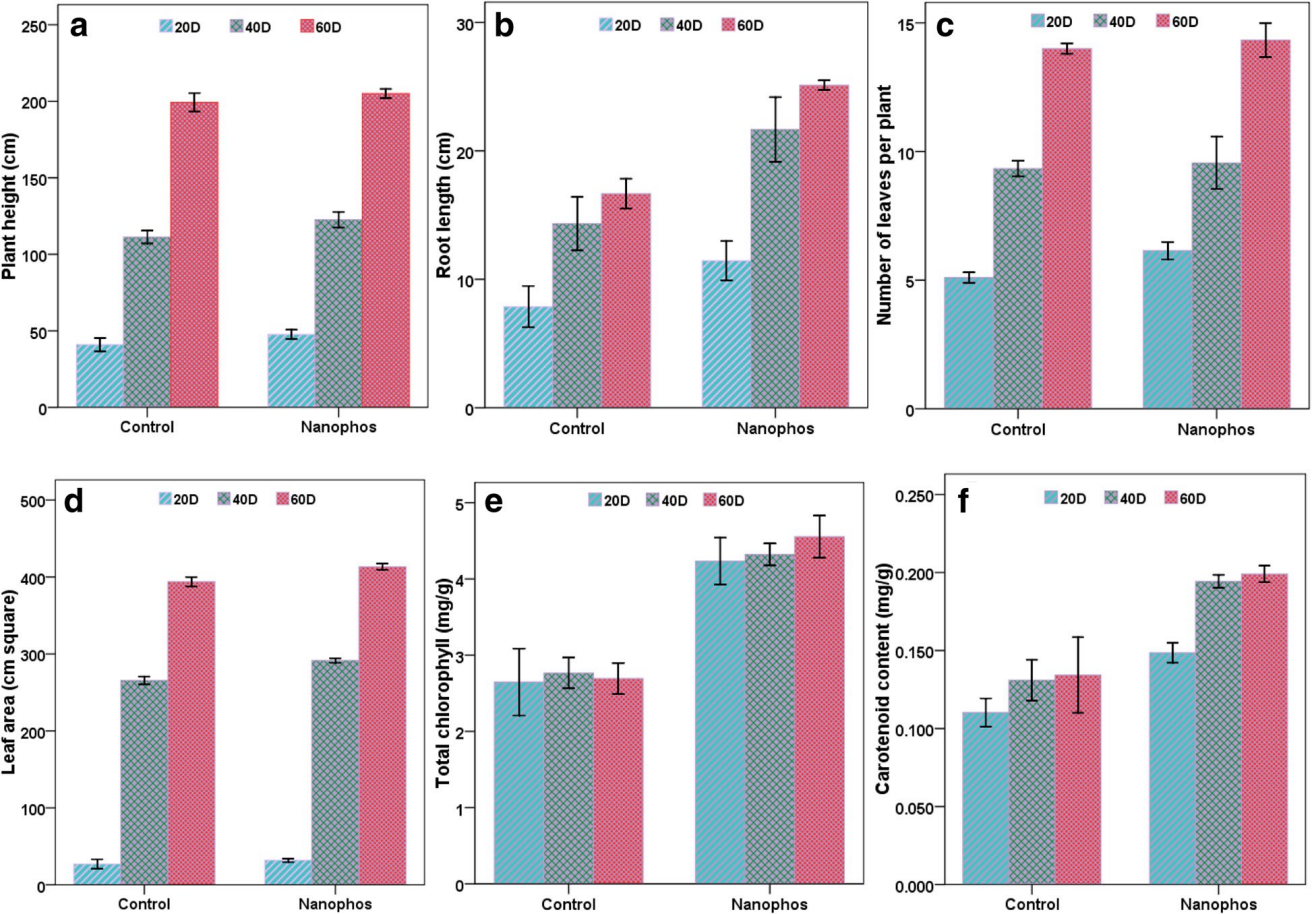
Effect of nanophos on agronomical and biochemical parameters of *Zea mays*

Four plants per plot (total 12 plants) were taken for the evaluation of agronomical and biochemical analysis. The data presented in Fig. 1 showed response of nanophos on plant height and number of leaves. 16.89 and 9.94% increase plant height were observed in nanophos treated soil in comparison to control after 20 and 40 days of sowing. Root length showed 53.76, 54.71 and 48.75% increase in nanophos treated plants and showing the *p* value less than *P* < 0.05 means statically different but moderately correlated to the control estimated using Spearman correlation test for plant height and root length (SM1). Overall enhancement in leaf area, leaf number and fresh/dry weight of shoot/root was observed nanophos treatment over control but not statically different having the p value greater than *P* > 0.05 (SM2).

### Effect on biochemical parameters

Data analysis clearly showed that there was significant increase in all photosynthetic pigments (total chlorophyll and carotenoid) of nanophos plants over control (Fig.1). Total chlorophyll content of maize leaves was found in 4.23, 4.32 and 4.55 mg g^− 1^ leaves in nanophos treated plants after 20, 40 and 60 days of sowing, least was observed in control which showed 2.62, 2.66 and 2.68 mg g^− 1^. Carotenoid content also followed the same pattern and showed 34.54, 48.09 and 51.90% increase in treated plants after 20, 40 and 60 DAS over control. The *p* value for chlorophyll and carotenoid content in nanophos treated plants was *P* < 0.05 statically different from the control after the 60 days and moderately correlated to each other shown in supplementary figure (SM3).

### Total sugar protein and phenolic content

**Fig. 2:**
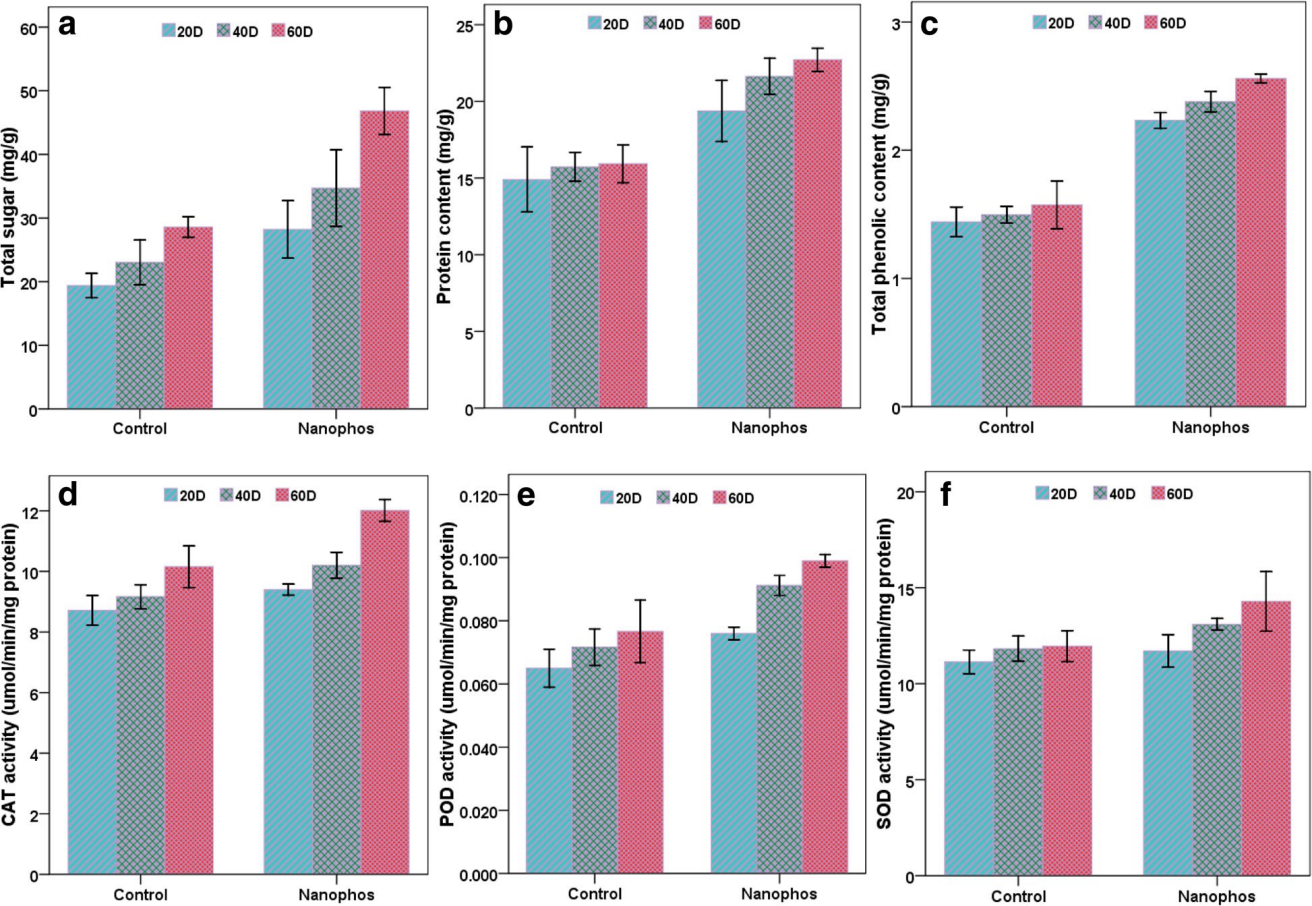
Effect of nanophos on biochemical parameters and antioxidant enzyme activity of *Zea mays*

Biochemical attributes were enhanced by nanophos treatment. Maximum sugar level was observed in maize plants treated with nanophos showed 45.76, 37.50 and 42.56% increase after 20, 40 and 60 DAS over control. Protein content in plant leaves was positively influenced by nanophos showing 30, 37.50 and 42.42% rise in protein content over control (Fig.2). Total phenolic content in treated plants showed 54.86, 59.73 and 63.05% increase in maize plants treated with nanophos over control. The *p* value for sugar, protein and phenols in nanophos treated plants was *P* < 0.05 statically different from the control after 40 and 60 days (SM4–5).

### Antioxidant enzymes

Increase in CAT activity was observed in nanophos treated plants showed 7.91, 11.23 and 18.32% increase over control. Same pattern was followed by POD and SOD activity in treated plants and showed 16.92, 28.16, 30.26% and 5.02, 10.74 and 19.28% increase in POD and SOD activity after 20, 40 and 60 DAS as compared to control (Fig.2). The *p* value for CAT activity in nanophos treated plants was *P* < 0.05 statically different from the control after 20 days no more effect was observed in POD and SOD activity shown in figure (SM5–6).

### Maize yield

**Fig. 3:**
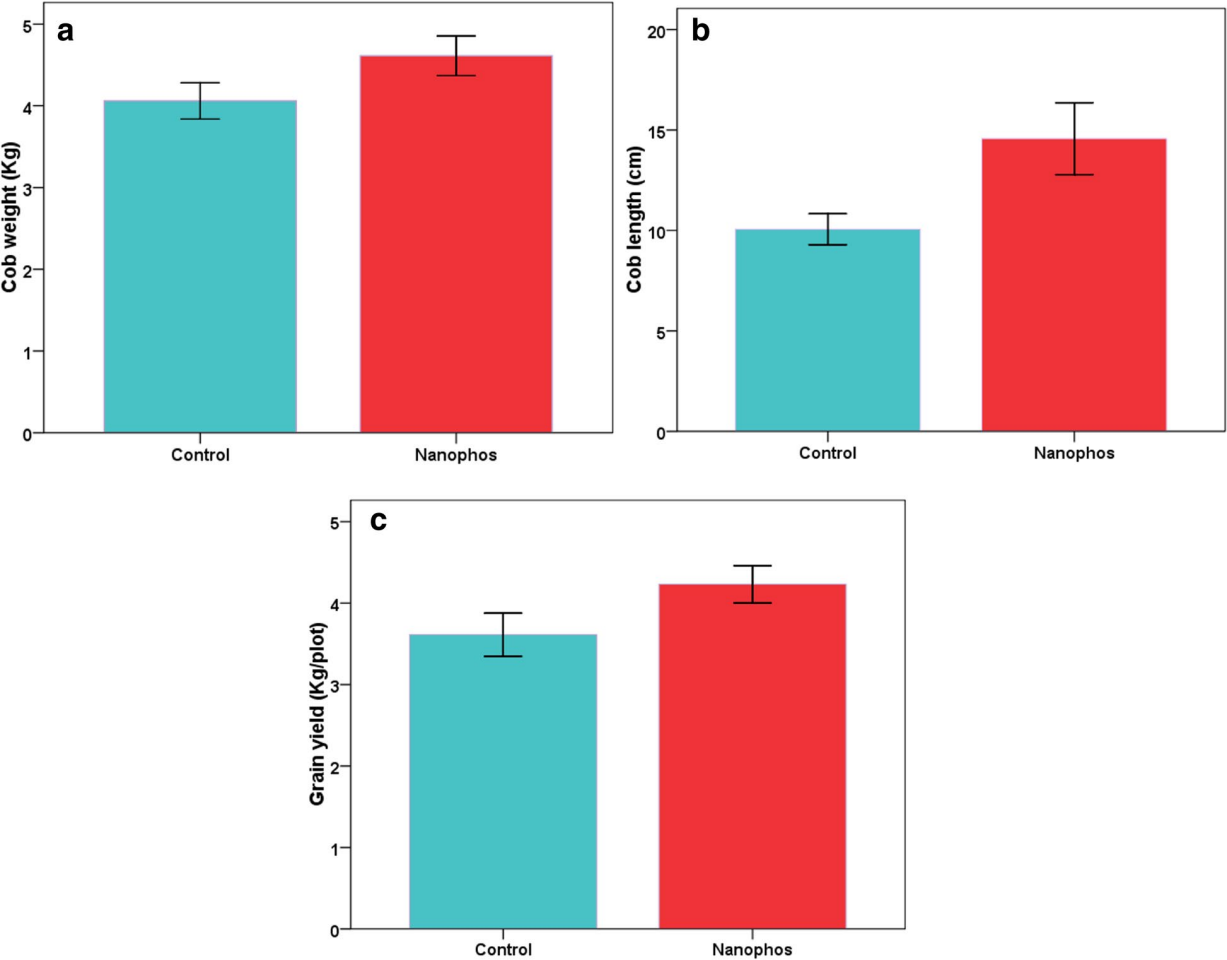
Effect of nanophos on yield of *Zea mays*

Increase in cob weight/length about 15 and 44.83% was observed in nanophos treatment over control. Grain yield/plot showed 17.17% increase in nanophos statically different over control estimated using correlation test. Increase in 100 grain weight of seeds in nanophos was observed and showed 8.34% increase as compared to control (Fig.3).

### Bacterial count of treated soil on different media

Total bacterial count in the soil treated with nanophos showed 2.28 × 10^6^, 2.30 × 10^6^ and 2.31 × 10^6^ CFU while control showed 2.16 × 10^6^, 2.20 × 10^6^ and 2.12 × 10^6^ count. Colony Forming Unit of *Azotobacter* in nanphos treated soil was 6.76 × 10^5^, 6.76 × 10^5^ and 7.20 × 10^5^ better than the control. Number of P and K solubilizers were found maximum in treated soil showed 1.00 × 10^6^ 1.01 × 10^6^, 1.02 × 10^6^ and 6.10 × 10^5^, 6.33 × 10^5^ and 6.23 × 10^5^ after 20, 40 and 60 DAS as compared to control (Table 1).

**Table 1:** Effect of Nanophos on bacterial count (CFU) and 16S rDNA gene of soil

Soil health parameters	Treatments	20D	40D	60D
**Total bacterial count**	Control	2.16 × 10^6^ ± 4.04	2.20 × 10^6^ ± 3.00	2.12 × 10^6^ ± 4.50
	Nanophos	2.28 × 10^6^ ± 3.00	2.30 × 10^6^ ± 4.50	2.31 × 10^6^ ± 5.68
**Nitrogen fixing bacteria**	Control	5.40 × 10^5^ ± 5.71	5.77 × 10^5^ ± 9.29	5.93 × 10^5^ ± 5.03
	Nanophos	6.76 × 10^5^ ± 5.29	7.06 × 10^5^ ± 4.04	7.20 × 10^5^ ± 3.58
**Phosphate solubilizers**	Control	8.00 × 10^5^ ± 5.56	8.53 × 10^5^ ± 4.72	8.60 × 10^5^ ± 3.00
	Nanophos	1.00 × 10^6^ ± 3.21	1.01 × 10^6^ ± 4.21	1.02 × 10^6^ ± 3.21
**Potassium solubilizers**	Control	5.20 × 10^5^ ± 3.05	5.40 × 10^5^ ± 2.64	5.50 × 10^5^ ± 2.00
	Nanophos	6.10 × 10^5^ ± 3.60	6.33 × 10^5^ ± 4.50	6.23 × 10^5^ ± 5.85
**16S rDNA abundance**	Control	4.78 × 10^5^ ± 1.29 × 10^2^	4.35 × 10^5^ ± 1.30 × 10^2^	4.23 × 10^4^ ± 1.14 × 10^2^
	Nanophos	6.91 × 10^6^ ± 1.10 × 10^2^	3.78 × 10^6^ ± 1.05 × 10^2^	1.98 × 10^7^ ± 1.20 × 10^2^

### Soil enzyme activities

**Fig. 4:**
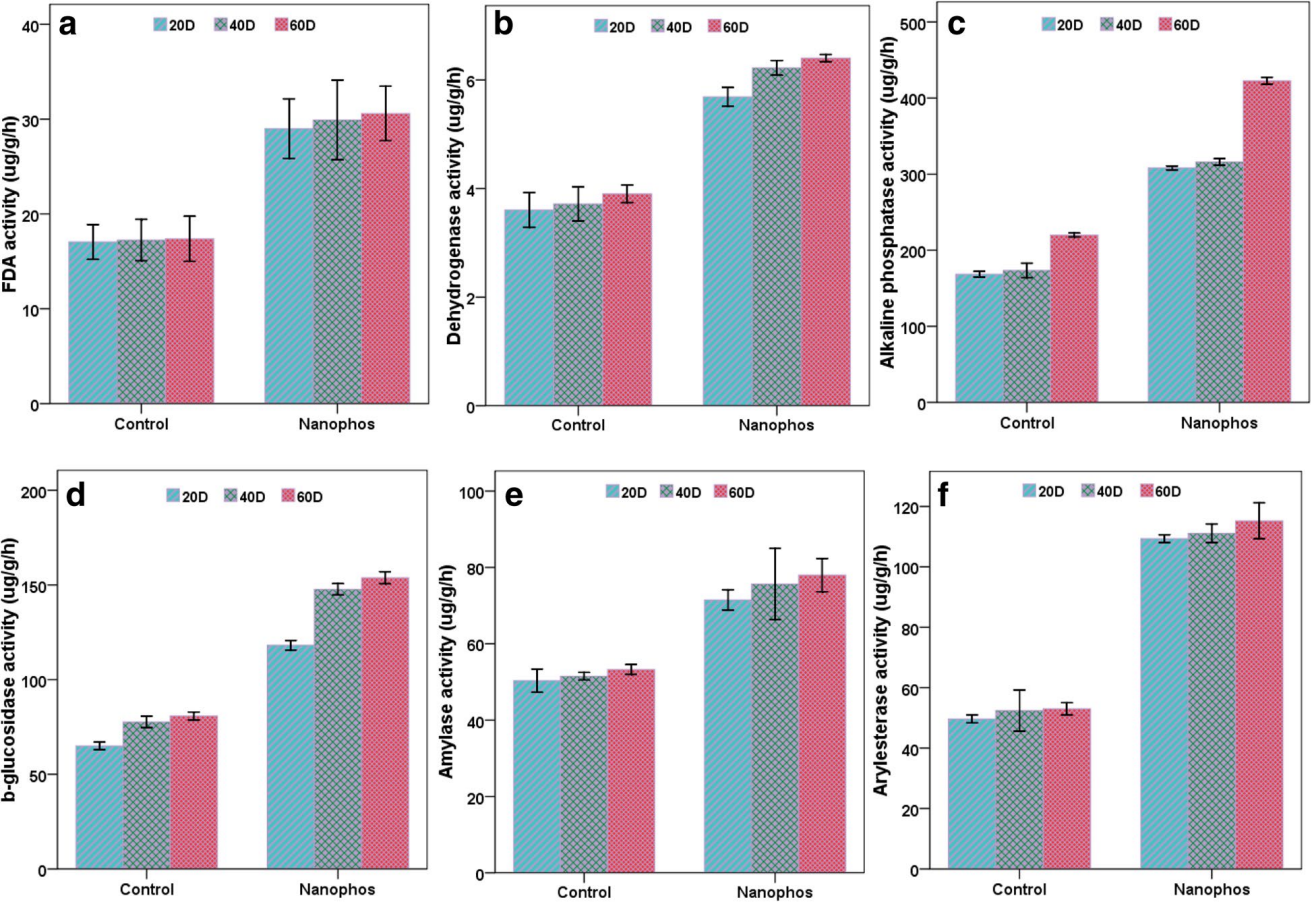
Effect of nanophos on soil enzyme activities after 20, 40 and 60 days of sowing

FDA acts as a substrate for the three enzymes protease, lipase and esterase, thus can be used as an indicator to check the activity of these enzymes. Nanophos had highest FDA hydrolysis activity (29, 29.91 and 30.58 μg fluorescein g^− 1^ h^− 1^) which was more than control. Activity of dehydrogenase was 5.69, 6.22 and 6.41 μg TPF g^− 1^ h^− 1^ was observed in nanophos treated soil better than the control (3.61, 3.72 and 3.90 μg TPF g^− 1^ h^− 1^). Phosphatase activity in nanophos treated soil was 308.16, 316.16 and 422.83 μg h^− 1^ higher as compared to control. β-glucosidase activity was 118.16, 147.66 and 153.83 μg h^− 1^ in nanophos treated soil. Amylase enzyme activity was 71.50, 75.66 and 78 μg h^− 1^ in nanophos, while control showed (50.33, 51.5 and 53.26 μg h^− 1^). Arylesterase activity was 109.33, 111.11 and 115.22 μg h^− 1^ in nanophos treated soil which was twofold increases over control (Fig. 4). The *p* value for above enzymes 0.03, 0.02, 0.04 after 60 days of the experiment in nanophos treated plants was *P* < 0.05 statically different from the control (SM7–9).

### qPCR analysis of 16S rDNA

Change in Copy number of 16S rDNA per gram soil sample was evaluated using qPCR and the values are expressed as copy number of the bacterial community under various treatments (Table 1). Gradual increase in 16S rDNA copy number up to 60 days of the experiment was observed in nanophos treated soil. Abundance of 16S rDNA was 4.78 × 10^5^, 4.35 × 10^5^, 4.35 × 10^5^ and 6.91 × 10^6^, 3.78 × 10^6^ and 1.98 × 10^7^ in control and nanophos treated soil respectively. The *p* value for in nanophos treated plants was *P* < 0.05 statically different from the control.

### Soil protein

It was observed that treated soil has 16 and 8 prominent bands of nearly 10, 13, 15, 16, 35, 40, 55, 70 and 100 kDa (lane 1,2) after 20 and 60 DAS, while in control soil only 5 bands were present (lane 3,4). Intense bands were observed in nanophos soil as compared to control (SM10).

## Discussion

Biofertilizers have been shown to beneficial effects on crop plant physiological action as well as soil health. Under field conditions, nanophos had a positive impact on plant and soil health parameters of *Zea mays*. Nanophos improved the ability of plants to absorb vital nutrients, as well as seed germination, grain maturity and crop yield. Influence of nanophos on agronomical, biochemical and yield of maize unravelling the growth promoting properties and can be useful in agricultural field. Phosphate solubilising bacteria increase the seed germination, plant/root length, leaf area and number of leaves. This may be due to the dissolving the insoluble phosphate compounds, production of phytohormones and enhancing the availability to plants by releasing organic acids and enzymes [37–39]. Biofertilizers along with nanocompounds are reported to enhance the seed germination and plant health productivity by nutrient cycling, production of plant hormones and solubilisation of different minerals [40, 41]. Combination of *Bacillus megatarium, Paenibacillus polymyxa* and *Rhizobium* promoted the shoot/root length and biomass of common bean plants due to increase the P availability which helps in growth and expansion of roots [42]. *Bacillus mucilaginous* and *B. megaterium* increase the phosphorus availability and chilli pepper yield in calcareous soil [43]. *Bacillus* spp. with phosphate fertilizer increased the yield of sugarcane under pot trial [44]. Application of nano fertilizers improved the rice yield reported by Valojai et al. [45]. Pallegrini et al. [46] reported that consortium of *Gluconacetobacter diazotrophicus* and *Burkholderia ambifaria* improved the soil nutrient status, total chlorophyll, growth and yield of onion plants. Enhanced level of antioxidant enzymes in nanophos treated plants such as CAT, POD and SOD. These enzymes can act as plant growth regulators and induce plant resistance towards phytopathogens. Nanophosphorus combination with PSB increased the total chlorophyll, SOD, CAT activity and yield of *Phaseolus vulgaris* significantly as compared to control in calcareous soil [47]. *Bacillus amyloliquefaciens* and *Paraburkholderia fungorum* increased the strawberry fruit growth, yield and antioxidant contents [48].

Soil NPK solubilising bacterial population also improved in nanophos treated soil. This is due to the positive impact of nanophos on bacterial community which involved in mineralization of P and other minerals in soil [49]. Rhizobial inoculants improved the heterotropic and P solubilizers count, higher dehydrogenase and urease activity under rye grass cultivation [50]. Biofertilizers application such as *Bacillus megaterium* increases the phosphorus content up to 39.7% [51, 52]. *Bacillus* sp. and *Pseudomonas taiwanensis* having the PGPR activities enhanced soil dehydrogenase, FDA, alkaline phosphatase activity, NPK solubilising population in soil and yield of maize plant under field condition [53, 54].

Soil enzymes are too susceptible and can be used as an indicator to analyse soil health. Different soil enzymes were significantly improved in treated soil indicating no negative impact of nanophos on soil health. Kumari et al. [55] found that nanophos (2 ml L^− 1^) improved the soil enzyme activities like urease, dehydrogenase and alkaline phosphatase under groundnut cultivation over control. Application of *B. aryabhattai* and *Pseudomonas auricularis* improved NPK content of the rhizospheric soil and photosynthetic efficiency in *Camellia oleifera* plants [56]. PGPR involved in enhancement of soil beneficial community and enhanced the soil enzyme activities [57, 58]. Application of *Bacillus* spp. improved maize growth and soil fertility by improving the nutrient status of the soil [59–61]. PSB increased the urease and dehydrogenase activity [62]. *Pseudomonas, Paraburkholderia* and *Ochrobactrum* (PSB) significantly improved the nutrient status and soil enzyme activities of the soil under Chinese fir seedlings [63]. Pyrosequencing approaches revealed that application of PSB on *Ulmus Chenmoui* favoured the bacterial population to certain extent and improved soil fertility [64, 65]. Ren et al. [66] reported that *Bacillus megatherian* along with biochar increase the NPK concentration and soil urease activity. Soil NPK content and phosphatase activity were enhanced when treated with PSB under wheat cultivation [67]. *Bacillus* spp. improved the *Proteobacteria, Chloroflexi* and *Bacteriodetes* population and improved the soil physicochemical properties under acid mine drainage [68].

Proteomics is a valuable method for inspecting the variation in protein profiles of microbes. A minor alteration in the environment can change the amount and expression of proteins in microbes. Soil protein assessed the organically bound nitrogen that can be mineralized using microbes and can be accessible towards plants. Proteomic divergence of soil is also a sign of multifaceted microbial dynamics. The majority of the extracellular enzymes secreted by microbes such as proteases, alkaline phosphatases, lipases and cellulases comes under the range of 20–200 kDa [69]. Alkaline phosphatase and glucosidase are essential for nutrient cycling and have a molecular weight of 80–90 kDa [70]. The banding pattern on the gel was better in treated sample, indicating a positive association between soil enzymes and soil microbes.

## Conclusion and recommendations

According to the findings of this study, nanophos has positive impact on maize agronomical, biochemical parameters, antioxidant enzymes, yield of maize and soil microbial population in maize crop. Nanophos treatment indicates good response towards total bacterial, NPK solubilising bacteria, soil enzymes and soil protein. It can be a potential way to enhance nutrient use efficiency in soil and can be a good alternative to agrochemicals used in the agricultural fields. However, more research is required in diverse environment to know the precise mechanisms of nanophos .

## Supplementary Information

**Additional file 1 **: **SM1**. Correlation analysis in control and nanophos treated sample after 20, 40 and 60 days. The darker the square is, the greater the *P*-value is. The values are calculated through Spearman analysis. **SM2**. Correlation analysis in control and nanophos treated sample after 20, 40 and 60 days. The darker the square is, the greater the *P*-value is. The values are calculated through Spearman analysis. **SM3**. Correlation analysis in control and nanophos treated sample after 20, 40 and 60 days. The darker the square is, the greater the *P*-value is. The values are calculated through Spearman analysis. **SM4**. Correlation analysis in control and nanophos treated sample after 20, 40 and 60 days. The darker the square is, the greater the *P*-value is. The values are calculated through Spearman analysis. **SM5**. Correlation analysis in control and nanophos treated sample after 20, 40 and 60 days. The darker the square is, the greater the *P*-value is. The values are calculated through Spearman analysis. **SM6**. Correlation analysis in control and nanophos treated sample after 20, 40 and 60 days. The darker the square is, the greater the *P*-value is. The values are calculated through Spearman analysis. **SM7**. Correlation analysis in control and nanophos treated sample after 20, 40 and 60 days. The darker the square is, the greater the *P*-value is. The values are calculated through Spearman analysis. **SM8**. Correlation analysis in control and nanophos treated sample after 20, 40 and 60 days. The darker the square is, the greater the *P*-value is. The values are calculated through Spearman analysis. **SM9**. Correlation analysis in control and nanophos treated sample after 20, 40 and 60 days. The darker the square is, the greater the *P*-value is. The values are calculated through Spearman analysis. **SM10**. SDS-PAGE photograph of soil protein in nanophos treated and control: Lane 1 and 2: nanophos treated soil after 20 and 60 days, Lane 3 and 4: control soil protein after 20 and 60 days of sowing, Lane 5: Prestained protein Ladder (10–180 kDa).

## Data Availability

All the relevant data are within the paper.

## Abbrevations

PSB: Phosphate solubilizing bacteria
CAT: Catalase
POD: Peroxidase
SOD: Superoxide dismutase
FDA: Fluorescein diacetate
SDS: Sodium dodecyl sulphate
