# Supplementary material for: Impact of nanophos in agriculture to improve functional bacterial community and crop productivity
Source: BMC Plant Biol. 2021 Nov 8;21:519. doi: 10.1186/s12870-021-03298-7 (PMC8573984; doi:10.1186/s12870-021-03298-7)
Supplement: Supplementary file 1 — Additional file 1 : SM1. Correlation analysis in control and nanophos treated sample after 20, 40 and 60 days. The darker the square is, the greater the P-value is. The values are calculated through Spearman analysis. SM2. Correlation analysis in control and nanophos treated sample after 20, 40 and 60 days. The darker the square is, the greater the P-value is. The values are calculated through Spearman analysis. SM3. Correlation analysis in control and nanophos treated sample after 20, 40 and 60 days. The darker the square is, the greater the P-value is. The values are calculated through Spearman analysis. SM4. Correlation analysis in control and nanophos treated sample after 20, 40 and 60 days. The darker the square is, the greater the P-value is. The values are calculated through Spearman analysis. SM5. Correlation analysis in control and nanophos treated sample after 20, 40 and 60 days. The darker the square is, the greater the P-value is. The values are calculated through Spearman analysis. SM6. Correlation analysis in control and nanophos treated sample after 20, 40 and 60 days. The darker the square is, the greater the P-value is. The values are calculated through Spearman analysis. SM7. Correlation analysis in control and nanophos treated sample after 20, 40 and 60 days. The darker the square is, the greater the P-value is. The values are calculated through Spearman analysis. SM8. Correlation analysis in control and nanophos treated sample after 20, 40 and 60 days. The darker the square is, the greater the P-value is. The values are calculated through Spearman analysis. SM9. Correlation analysis in control and nanophos treated sample after 20, 40 and 60 days. The darker the square is, the greater the P-value is. The values are calculated through Spearman analysis. SM10. SDS-PAGE photograph of soil protein in nanophos treated and control: Lane 1 and 2: nanophos treated soil after 20 and 60 days, Lane 3 and 4: control soil protein after 20 and 60 days of sowing [file 12870_2021_3298_MOESM1_ESM.docx]

**Supplementary Material**


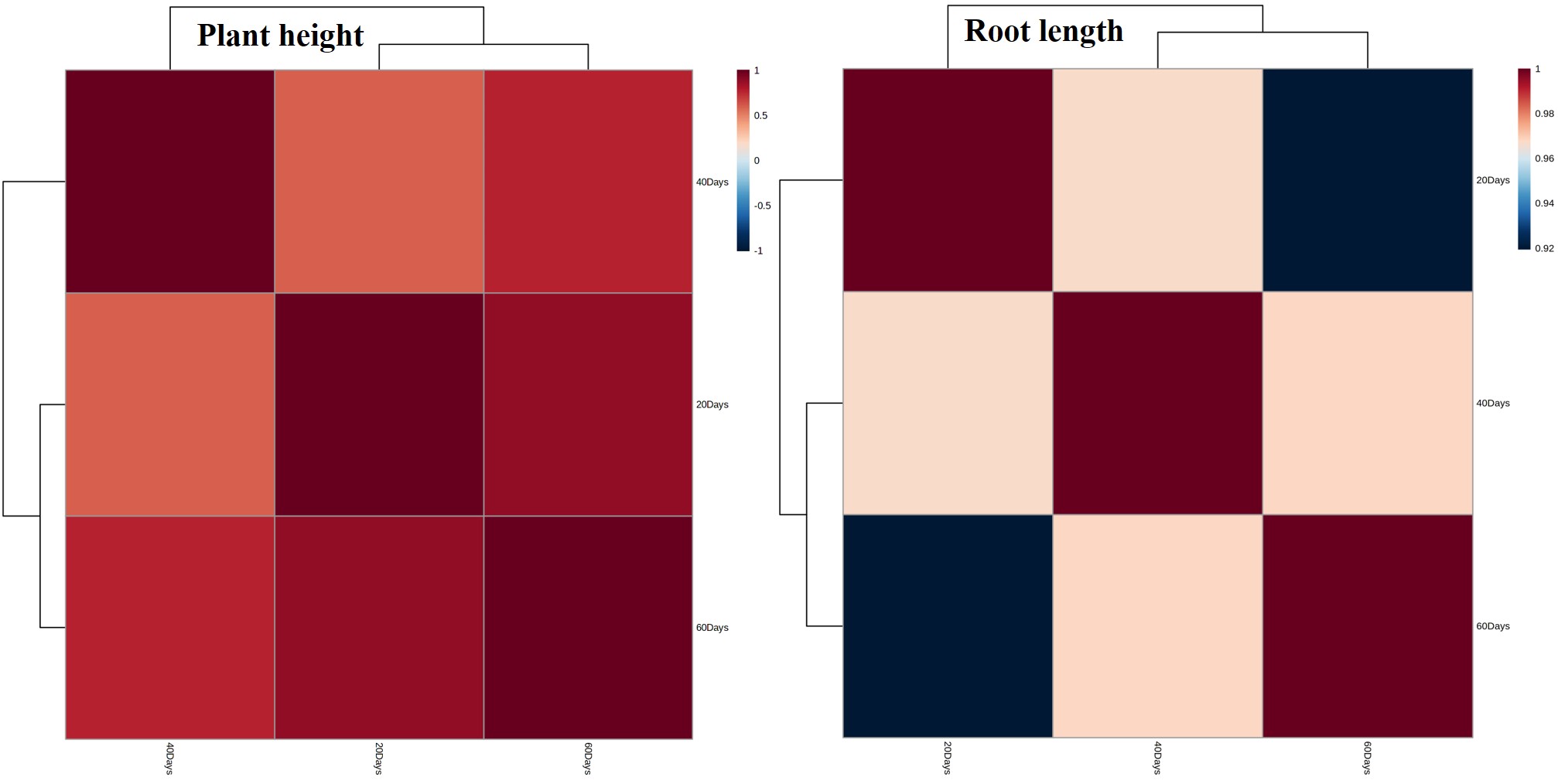


**SM1:** Correlation analysis in control and nanophos treated sample after 20,40 and 60 days. The darker the square is, the greater the P-value is. The values are calculated through Spearman analysis.


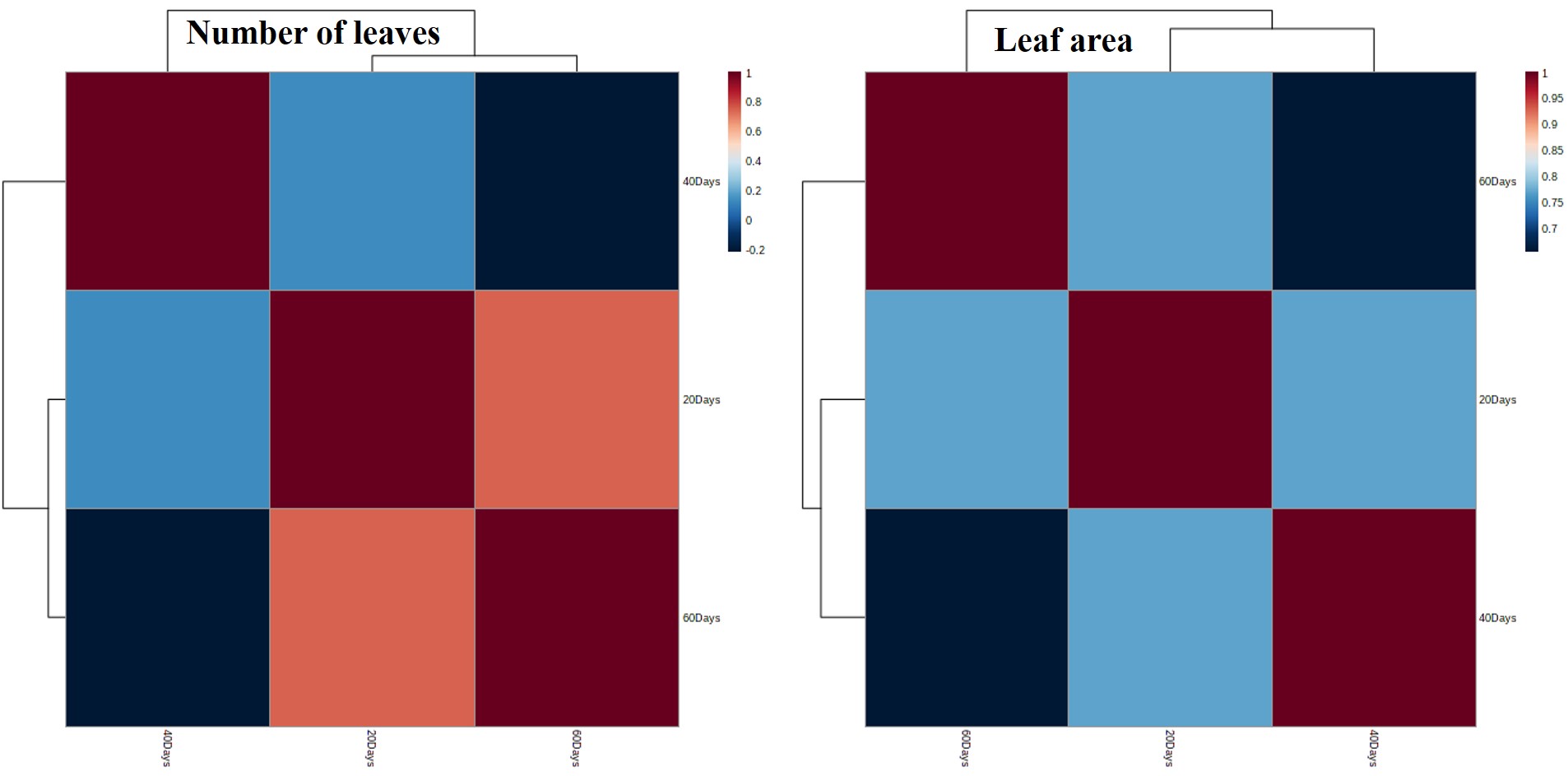


**SM2:** Correlation analysis in control and nanophos treated sample after 20,40 and 60 days. The darker the square is, the greater the P-value is. The values are calculated through Spearman analysis.


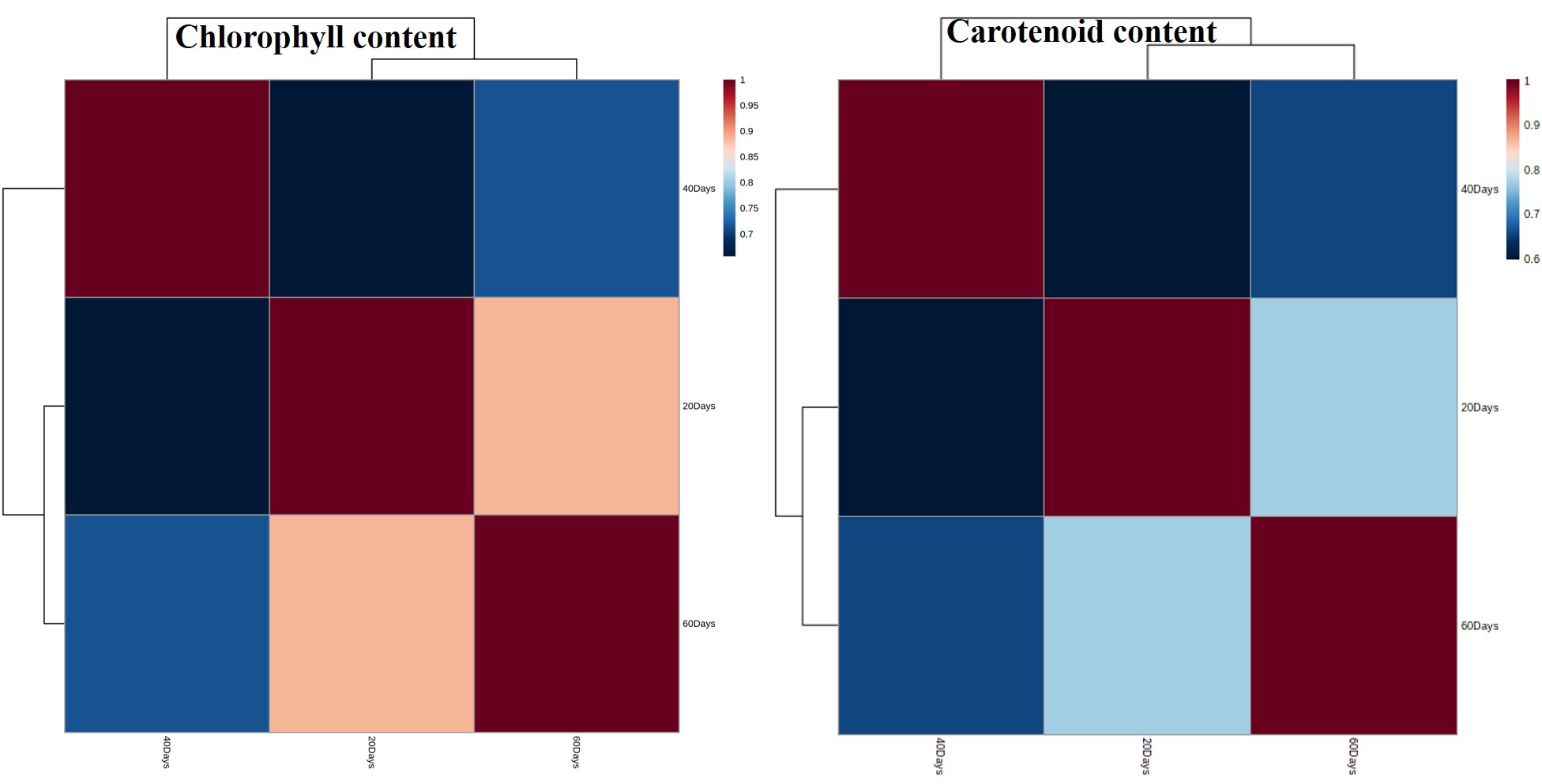


**SM3:** Correlation analysis in control and nanophos treated sample after 20,40 and 60 days. The darker the square is, the greater the P-value is. The values are calculated through Spearman analysis.


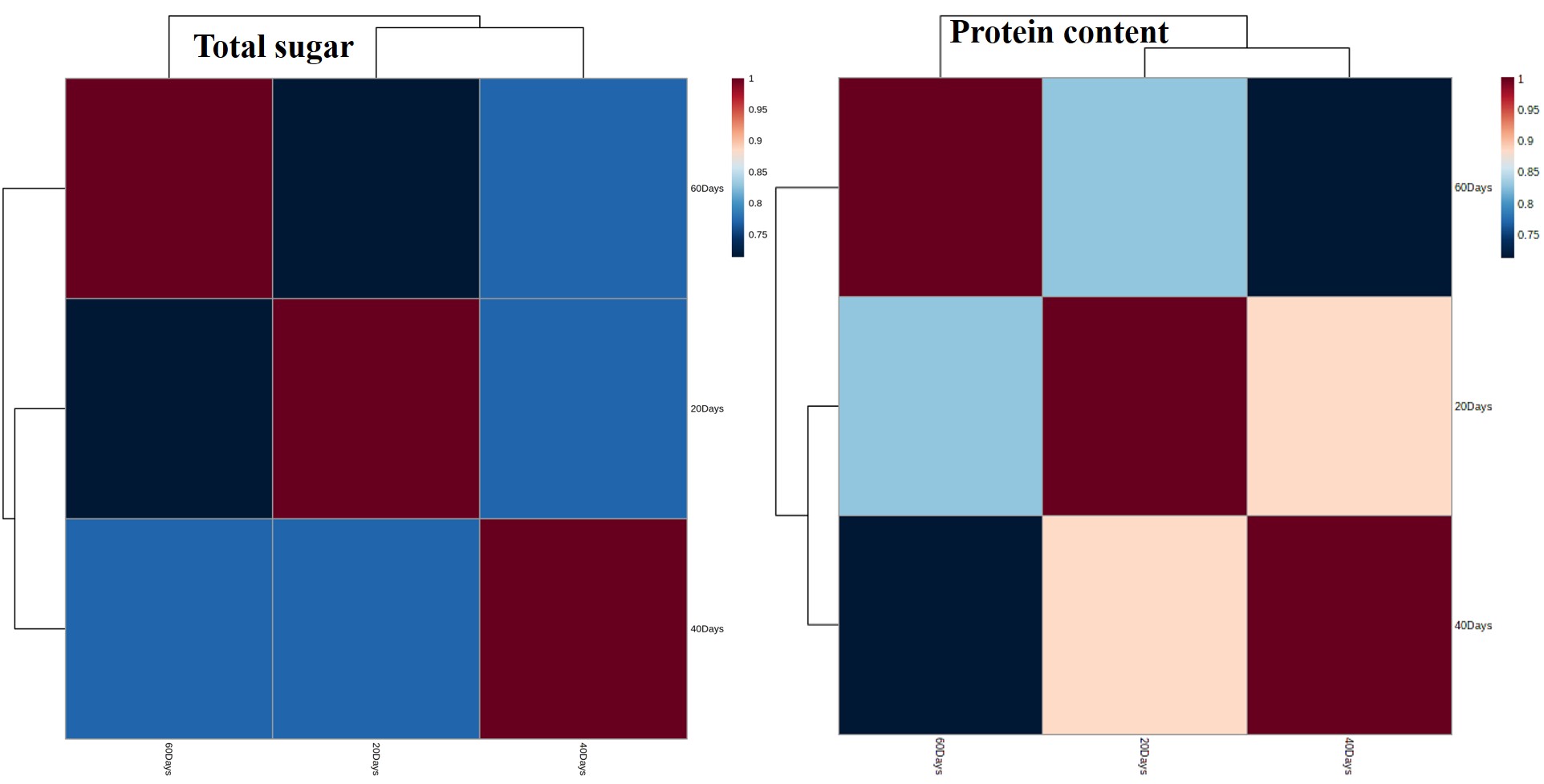


**SM4:** Correlation analysis in control and nanophos treated sample after 20,40 and 60 days. The darker the square is, the greater the P-value is. The values are calculated through Spearman analysis.


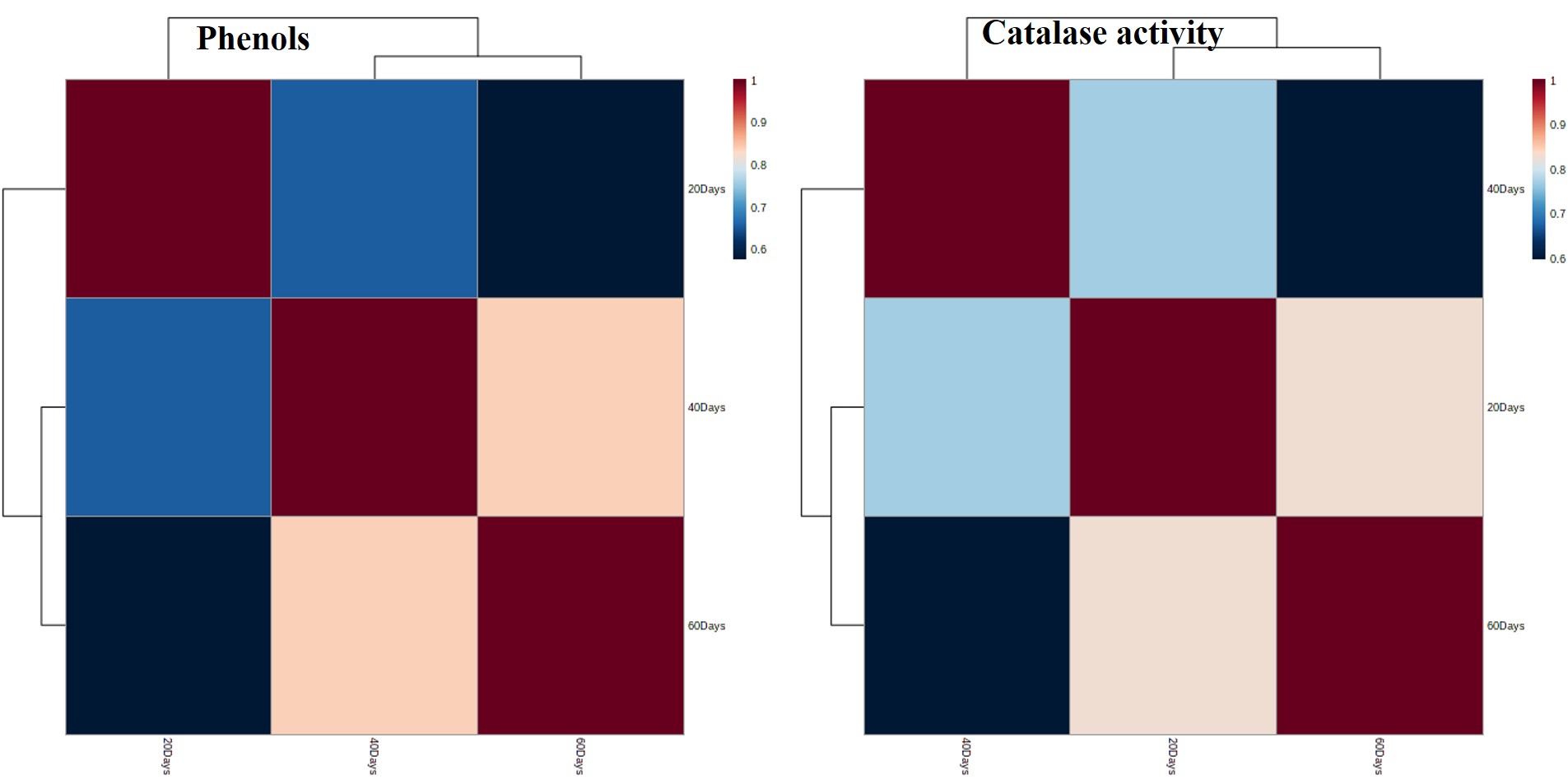


**SM5:** Correlation analysis in control and nanophos treated sample after 20,40 and 60 days. The darker the square is, the greater the P-value is. The values are calculated through Spearman analysis.


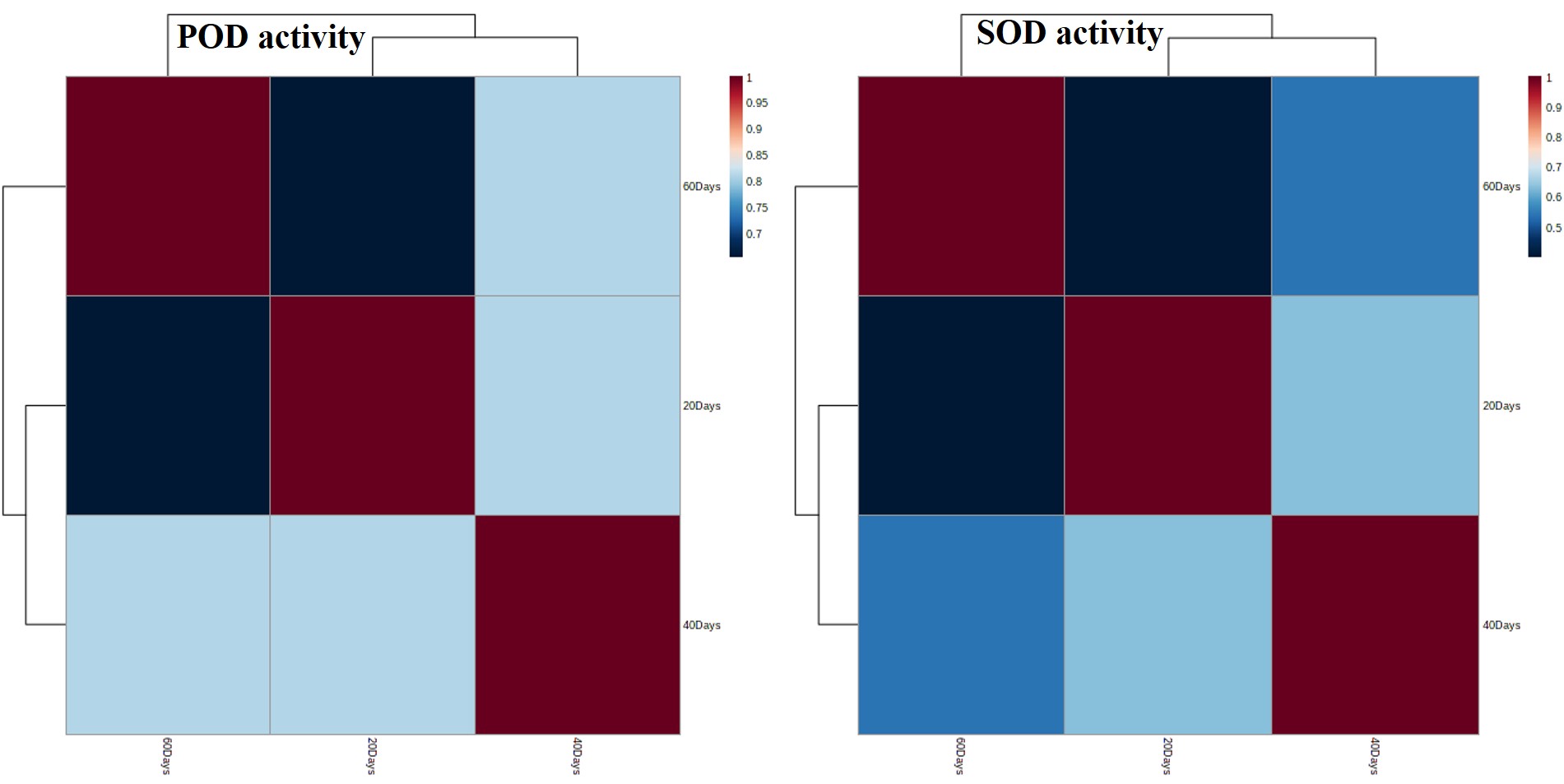


**SM6:** Correlation analysis in control and nanophos treated sample after 20,40 and 60 days. The darker the square is, the greater the P-value is. The values are calculated through Spearman analysis.


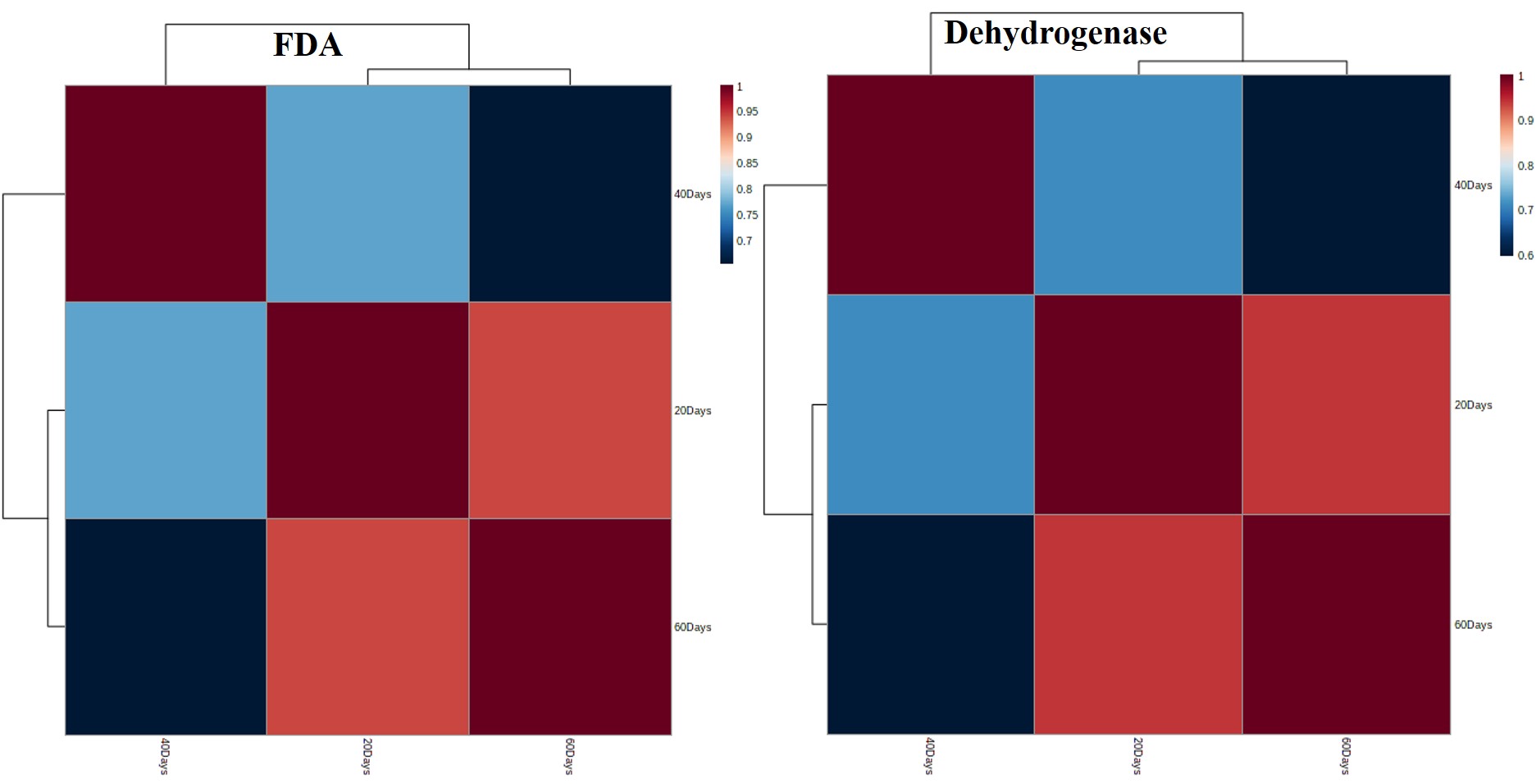


**SM7:** Correlation analysis in control and nanophos treated sample after 20,40 and 60 days. The darker the square is, the greater the P-value is. The values are calculated through Spearman analysis.


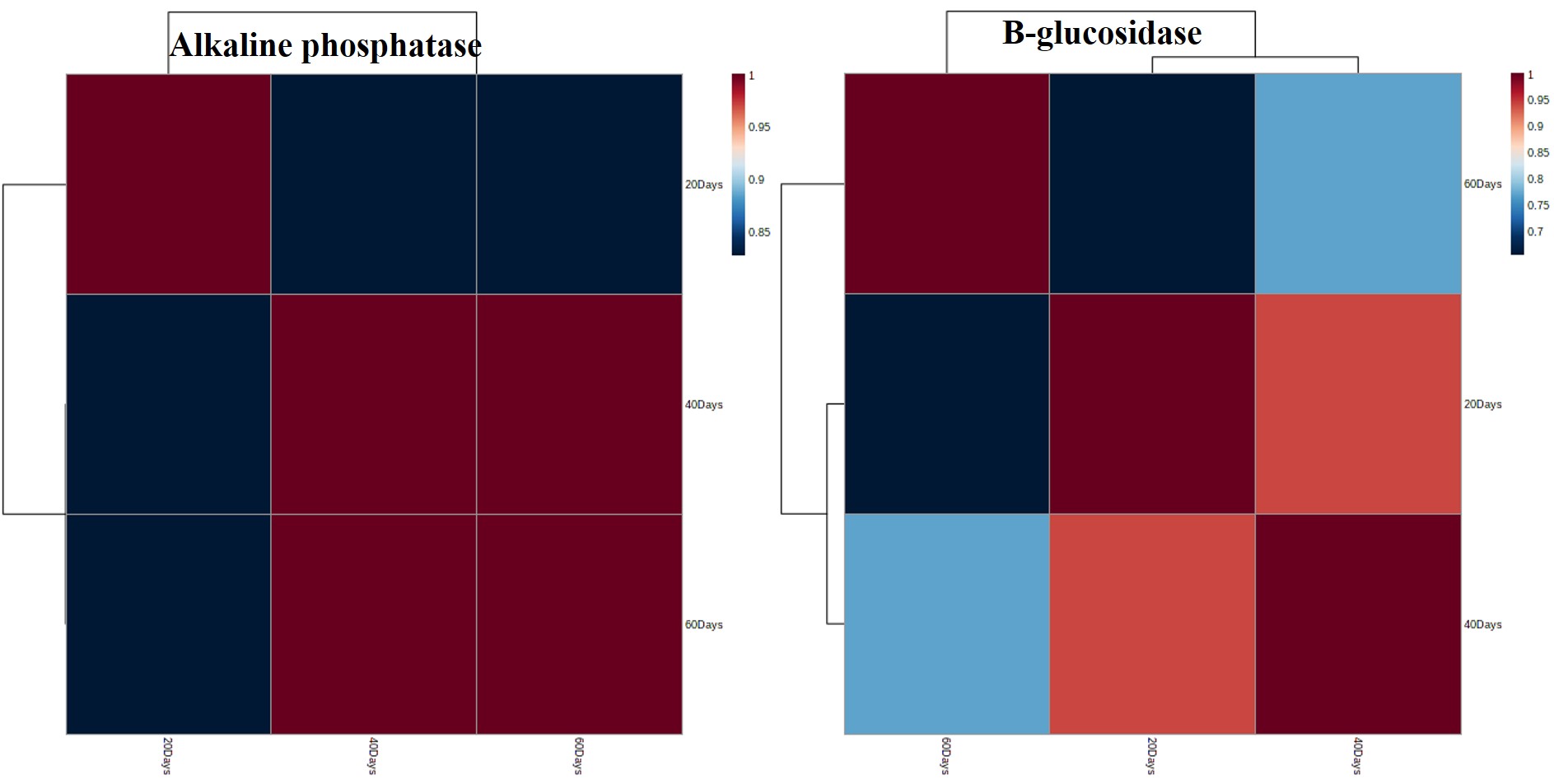


**SM8:** Correlation analysis in control and nanophos treated sample after 20,40 and 60 days. The darker the square is, the greater the P-value is. The values are calculated through Spearman analysis.


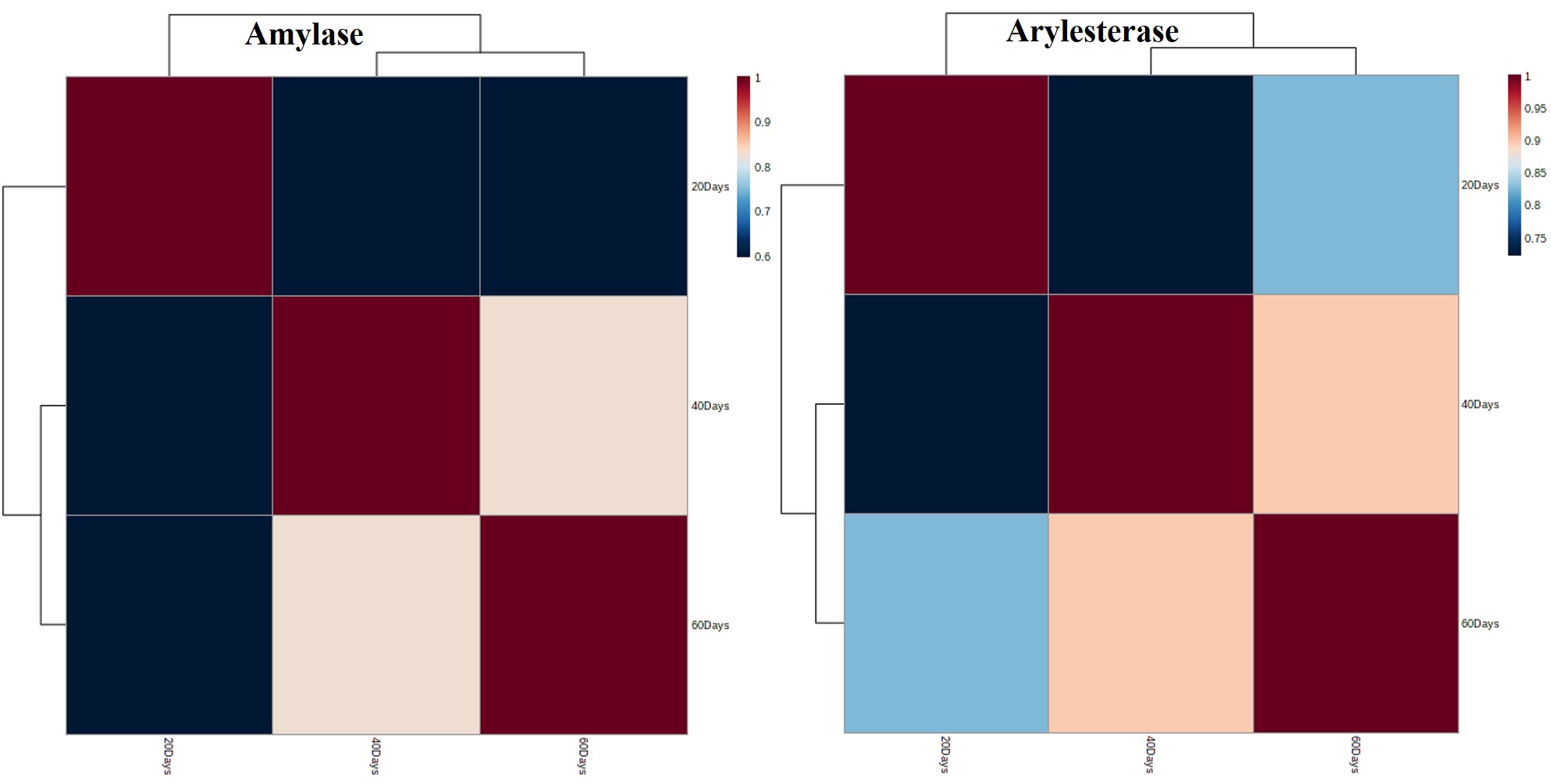


**SM9:** Correlation analysis in control and nanophos treated sample after 20,40 and 60 days. The darker the square is, the greater the P-value is. The values are calculated through Spearman analysis.


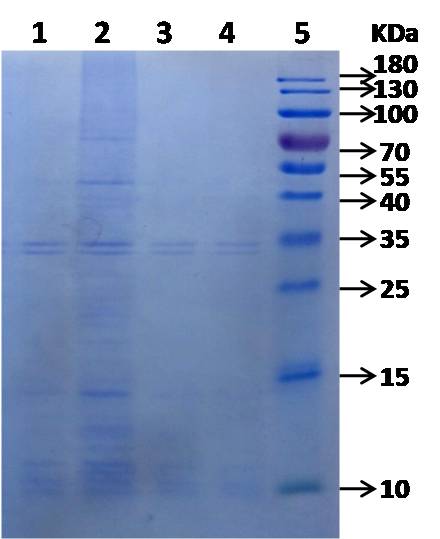

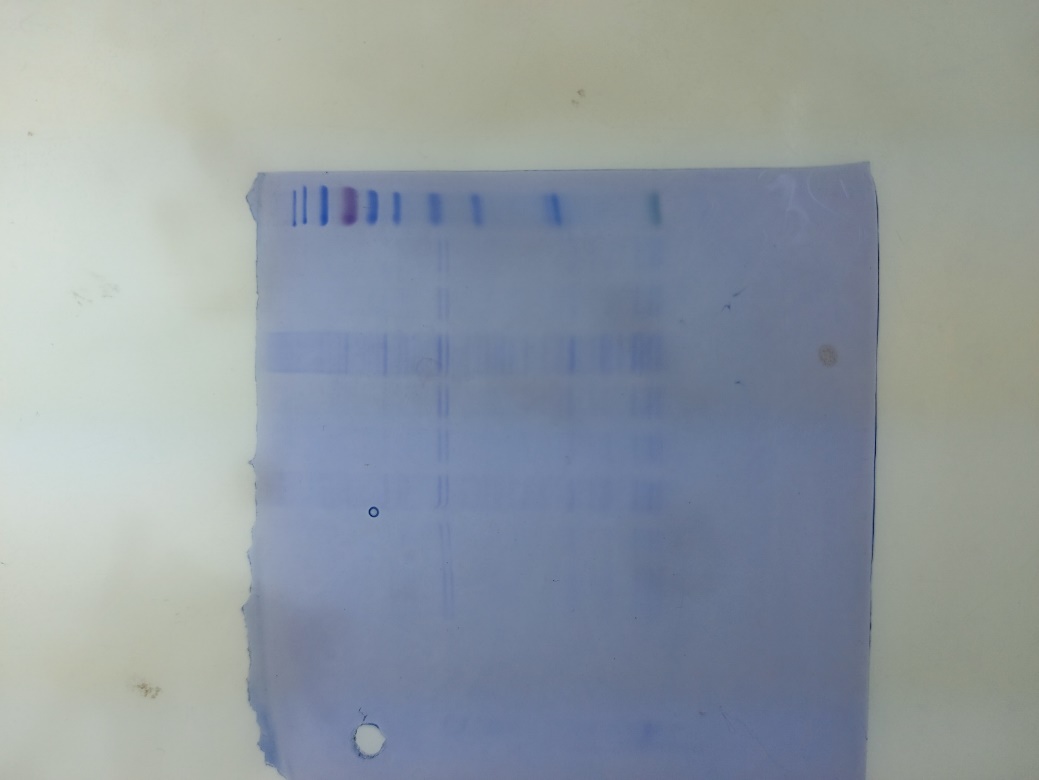


**SM10:** SDS-PAGE photograph of soil protein in nanophos treated and control: Lane 1 and 2: nanophos treated soil after 20 and 60 days, Lane 3 and 4: control soil protein after 20 and 60 days of sowing, Lane 5: Prestained protein Ladder (10-180 kDa).
